# Water-Based Suspensions of Iron Oxide Nanoparticles with Electrostatic or Steric Stabilization by Chitosan: Fabrication, Characterization and Biocompatibility

**DOI:** 10.3390/s17112605

**Published:** 2017-11-13

**Authors:** Galina V. Kurlyandskaya, Larisa S. Litvinova, Alexander P. Safronov, Valeria V. Schupletsova, Irina S. Tyukova, Olga G. Khaziakhmatova, Galina B. Slepchenko, Kristina A. Yurova, Elena G. Cherempey, Nikita A. Kulesh, Ricardo Andrade, Igor V. Beketov, Igor A. Khlusov

**Affiliations:** 1 Departamento de Electricidad y Electrónica and BCMaterials, Universidad del País Vasco UPV-EHU, 48080 Bilbao, Spain; 2 Institute of Natural Sciences and Mathematics, Ural Federal University, Ekaterinburg 620002, Russia; safronov@iep.uran.ru (A.P.S.); tyukov.sergey@mail.ru (I.S.T.); kuleshnik@list.ru (N.A.K.); 3 Laboratory of Immunology and Cell Biotechnology, I. Kant Baltic Federal University, Kaliningrad 23601, Russia; larisalitvinova@yandex.ru (L.S.L.); vshupletsova@mail.ru (V.V.S.); hazik36@mail.ru (O.G.K.); kristina_kofanova@mail.ru (K.A.Y.); 4 Institute of Electrophysics, Ural Division RAS, Ekaterinburg 620016, Russia; igor.beketov@mail.ru; 5 Department of Physical and Analytical Chemistry, National Research Tomsk Polytechnic University, Tomsk 634050, Russia; slepchenkogb@mail.ru (G.B.S.); cherempey@mail.ru (E.G.C.); 6 Advanced Research Facilities (SGIKER), Universidad del País Vasco UPV-EHU, 48080 Bilbao, Spain; ricardo.andrade@ehu.eus; 7 Department of Experimental Physics, National Research Tomsk Polytechnic University, Tomsk 634050, Russia; khlusov63@mail.ru

**Keywords:** magnetic biosensors, iron oxide magnetic nanoparticles, chitosan, ferrofluids, human blood mononuclear leukocytes, morphofunctional response

## Abstract

Present day biomedical applications, including magnetic biosensing, demand better understanding of the interactions between living systems and magnetic nanoparticles (MNPs). In this work spherical MNPs of maghemite were obtained by a highly productive laser target evaporation technique. XRD analysis confirmed the inverse spinel structure of the MNPs (space group Fd-3m). The ensemble obeyed a lognormal size distribution with the median value 26.8 nm and dispersion 0.362. Stabilized water-based suspensions were fabricated using electrostatic or steric stabilization by the natural polymer chitosan. The encapsulation of the MNPs by chitosan makes them resistant to the unfavorable factors for colloidal stability typically present in physiological conditions such as pH and high ionic force. Controlled amounts of suspensions were used for in vitro experiments with human blood mononuclear leukocytes (HBMLs) in order to study their morphofunctional response. For sake of comparison the results obtained in the present study were analyzed together with our previous results of the study of similar suspensions with human mesenchymal stem cells. Suspensions with and without chitosan enhanced the secretion of cytokines by a 24-h culture of HBMLs compared to a control without MNPs. At a dose of 2.3, the MTD of chitosan promotes the stimulating effect of MNPs on cells. In the dose range of MNPs 10–1000 MTD, chitosan “inhibits” cellular secretory activity compared to MNPs without chitosan. Both suspensions did not caused cell death by necrosis, hence, the secretion of cytokines is due to the enhancement of the functional activity of HBMLs. Increased accumulation of MNP with chitosan in the cell fraction at 100 MTD for 24 h exposure, may be due to fixation of chitosan on the outer membrane of HBMLs. The discussed results can be used for an addressed design of cell delivery/removal incorporating multiple activities because of cell capability to avoid phagocytosis by immune cells. They are also promising for the field of biosensor development for the detection of magnetic labels.

## 1. Introduction

Magnetic ferrofluids (FFs) have been attracting increasing interest for technological and biomedical applications in recent years. A FF is a stable suspension of colloidal magnetic nanoparticles (MNPs) dispersed in a carrier liquid [1].Biomedical applications demand magnetic MNPs in the form of water-based ferrofluids or ferrogels [2,3]. The list of proposed applications has extended rapidly, counting with uses such as drug delivery carriers, contrast media for magnetic resonance imaging, magnetic labels for magnetic biosensing, thermal ablation and hyperthermia, thermal activation for drug release and others [4,5,6,7]. Asystematic comparative analysis of the morphofunctional response of different kinds of living cells to the presence of MNPs is still lackingbut a better understanding of the interactions of the living systems with MNPs and search for synergetic combinations “cell type-MNPs” would be beneficial [8]. The progress in the fabrication of the biocomposites is especially important for the field of magnetic biosensing because further development of the compact analytical devices in which a magnetic transducer converts a magnetic field variation into a change of frequency, current, voltage requires thoroughly characterized biological samples with well-known amount and distribution of MNPs [9,10].

The necessary condition for the biomedical application of the suspension of MNPs is the maintenance of its colloidal stability in the neutral range of pH and high ionic strength, inherent to biological tissues. There are two main possibilities to obtain stable colloidal suspensions of deaggregated nanoparticles: electrostatic and steric stabilization [9]. An increase of the ionic strength of the biological medium makes the electrostatic stabilization of the suspensions less effective. In a number of recent studies the natural polymer chitosan has been discussed as an effective electrosteric stabilizer [11,12]. 

**Figure 1:**
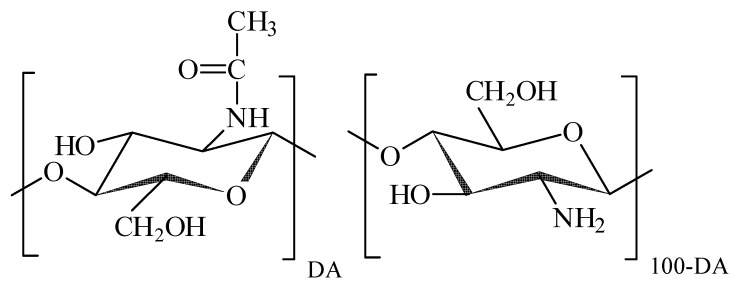
Chemical structure of chitosan monomer units: acetylated to the left, deacetylated to the right. DA is the degree of acetylation.

Chitosan is a polysaccharide produced by chemical modification of a natural polymer—chitin. The biocompatibility, biodegradability, immuno-stimulating features, high chemical reactivity of chitosan provides its extensive usage in a variety of biomedical and bioengineering applications [13]. Chitosan is a polyelectrolyte, whose typical molecular structure is shown in Figure 1. 

The macromolecular backbone is based on β-glucose units with hydroxyl residue in 2nd position substituted either by acetamide or by amine group. In the natural precursor of chitosan (chitin) all monomeric units contain acetamide moieties, which are partly or completely removed during chemical modification of chitin, which is known as deacetylation. The degree of acetylated units (DA, %) remained in the molecular structure is the characteristic feature of the chemical composition of chitosan, which governs its interaction with water and solutes. Amine groups in the deacetylated monomer units can be protonated and thus they can carry on positive electrical charge, which makes chitosan a polycation. The polycationic nature of chitosan favours its interaction with polyanionic biomacromolecules such as proteins and nucleic acids, which results in the formation of polyelecrolyte complexes. The formation and the stability of chitosan polyelectrolyte complexes depends on the DA, charge density, molecular weight, quality of a solvent, ionic strength, pH, and temperature [14]. The formation of chitosan—protein complexes takes place if both counterparts are ionized. Polyelectrolyte complexes with collagen, gelatin, and milk albumin are reported in literature [15].

Chitosan is widely tested as an efficient biocompatible stabilizer in the suspensions of nanoparticles of different chemical origin designed for biomedical applications [16]. The presence of both hydroxyl and amine residues in chitosan monomer unit enhance specific interaction of chitosan macromolecules with the surface of metal oxide MNPs and provide the stability of suspensions at low polymer concentration [17,18]. However, the majority of the reported data on the stabilization of the suspensions of MNPs by chitosan are related to acidic solutions in which chitosan is protonated and acts as a polycation. Meanwhile, the necessary condition for the biomedical application of the suspension of MNPs is the maintenance of its colloidal stability in the neutral range of pH and high ionic strength, inherent for biological tissues. In the neutral pH range as its monomer units are deprotonated, chitosan is not efficient as a stabilizer. In our recent works [19,20] we had introduced a route to overcome this limitation and provide the stabilization of iron oxide MNPs by chitosan in phosphate buffer saline (PBS, pH = 6.3), which is widely used in biomedical and bioengineering studies. 

This approach is based on the well-known feature of colloidal suspensions that their stability depends not only on the composition but also on the prior history of their preparation. First, suspensions of iron oxide MNPs electrostatically stabilized by sodium citrate were prepared. Sodium citrate is a very good stabilizer for iron oxide MNPs in water due to the adsorption of citrate anions onto the MNPs’ surface. It was shown earlier [21,22] that using citrate as an electrostatic stabilizer the suspension of individual iron oxide MNPs could be obtained. At the next step the electrostatically stabilized suspension was mixed with acidic chitosan solution. As chitosan was protonated and carried positive charges, it interacted with negatively charged MNPs electrostatically and adsorbed on their surface. Then PBS was added to deprotonate chitosan macromolecules and to make them collapse around MNPs. Thus the steric stabilization of MNPs resistant in high ionic strength was achieved. For the sake of comparison electro-statically stabilized water-based stable suspension of iron oxide nanoparticles from the same batch was also prepared using sodium citrate. We present our herein study on the fabrication, thorough characterization and biocompatibility of the suspensions of iron oxide MNPs electrostatically or sterically stabilized by chitosan tested for the case of human blood mononuclear leukocytes.

## 2. Experimental

### 2.1. Iron Oxide MNPs

Iron oxide MNPs were synthesized by laser target evaporation (LTE)—the method of high power physical dispersion of iron oxide in gas phase by laser irradiation. The details of the experimental procedure are given elsewhere [22,23]. LTE was performed using laboratory installation with Ytterbium (Yb) fiber laser with 1.07 μm wavelength. The laser operated in a pulsed regime with pulse frequency 4.85 KHz and pulse duration 60 μs. Average output power of irradiation was 212 W. The target pellet of 65 mm in diameter, 20 mm in height was pressed from commercial magnetite (Fe_3_O_4_) (Alfa Aesar, Ward Hill, MA, USA) powder (specific surface area 6.9 m^2^/g). The target was evaporated in a mixture of N_2_ and O_2_ in the volume ratio 0.79:0.21.

The X-ray diffraction (XRD) studies were performed with a DISCOVER D8 diffractometer (Bruker, Billerica, MA, USA) using Cu-K_α_ radiation (λ = 1.5418 Å), a graphite monochromator and a scintillation detector. The MNPs were mounted on a zero background silicon wafer placed in a sample holder. A fixed divergence and antiscattering slit were used. The quantitative analysis was done using the TOPAS-3 software with Rietveld full-profile refinement [24]. Transmission electron microscopy (TEM) was performed to evaluate themorphology of the MNPs (JEM2100, JEOL, Tokyo, Japan). The specific surface area of MNPs (S_sp_) was measured by the low-temperature adsorption of nitrogen (TriStar3000, Micromeritics, Norcross, GA, USA). 

Magnetic measurements of the hysteresis loops (M(H)) and thermomagnetic curves (ZFC-FC, see [23] for details) were carried out with a MPMSXL-7 SQUID-magnetometer (Quantum Design, San Diego, CA, USA) in the ±70 Oe field range. Thermomagnetic zero field cooled/field cooled (ZFC-FC) curves were obtained for the field of 100 Oe (5–300 K temperature range) for air-dry MNPs and MNPs dried from ferrofluids. Ferrofluids and biological samples were dried prior to measurement and polymer container contributions were carefully subtracted. 

### 2.2. Preparation of Ferrofluid and Suspension of MNPs Encapsulated by Chitosan

Before the preparation of the suspensions the iron oxide MNPs were dry-heat sterilized with a Binder FD53 device (Binder GmbH, Tuttlingen, Germany) at 180 °C for 1 h. Ferrofluid based on iron oxide MNPs was prepared in distilled water with the addition of an electrostatic stabilizer sodium citrate in 5 mM concentration. Suspension in an initial concentration 6% (by weight) was treated by ultrasound for 30 min using a CPX-750 processor (Cole-Parmer Instruments Corp., Vernon Hills, IL, USA) operated at 300 W power output. After the ultrasound treatment the suspension was centrifuged at 10,000 rpm for 5 min using Z383 centrifuge (Hermle, Hermle-labortechnik, Wehingen, Germany) equipped with a 218 rotor. Such a prepared suspension (electrostatically stabilized water-based ferrofluid) was designated FF1.

Encapsulation of MNPs by chitosan was performed using commercial chitosan SK-1000 (AlexinChem, Tula, Russia) with MW = 4.4 × 105 and DA = 30%. Chitosan was dissolved in 0.2M HCl to prepare 2% stock solution; the solution was kept 48 h at 25 °C for equilibration. Then chitosan stock solution was mixed with ferrofluid based on MNPs in 1:3 volume ratio and vigorously stirred. Then the pH of suspension was adjusted by the dropwise addition of PBS solution (in 1:2 volume ratios to suspension) under vigorous stirring. Such a prepared suspension (at a time electrostatically and sterically stabilized water-based ferrofluid) was designated the name FF2.

Hydrodynamic diameters of the MNPs and their aggregates were measured by dynamic light scattering (Zeta Plus particle size analyzer, Brookhaven Brookhaven Instruments Corp., Holtville, NY, USA). The electrokinetic zeta-potentials of the ferrofluids were measured by electrophoretic light scattering with the same instrument.

### 2.3. Cell Culture

The study of real biological samples (cells, unicelular organisms or tissues) has certain testing limitations when physical methods are employed. These limitations are caused by the wide variety of complex processes that exist in the living systems and the wide variety of morphologies of each particular sample. To make biophysical research withMNPs more effective, one must select model systems, with well known or most stable/typical properties. One of such model systems is human blood mononuclear leukocytes (HBMLs). HBMLs of health volunteer (Permit No. 4 from 23.10.2013 of Local Ethics Committee of Innovation park of Immanuel Kant Baltic Federal University, Russian Federation) were collected by venous blood gradient (ρ* = 1.077) Ficoll-Paque Premium (Sigma-Aldrich, St. Louis, MO, USA) centrifugation at 1500 rpm for 10 min. The HBMLs were twice washed by phosphate-buffered saline (рН 7.2) and resuspended in complete culture medium consisting of 90% RPMI-1640 (Sigma-Aldrich, St. Louis, MO, USA), 10% inactivated (for 30 min at 56 °C) fetal bovine serum (Sigma), and 0.3 mg/mLL-glutamine (Sigma). Viability was 90–95% for living cells unstained by 0.4% trypan blue. 

The HBMLs suspension was added into 24-well plates (Orange Scientific, Braine-l’Alleud, Belgium) with a final concentration of 2 × 10^6^ viable cells per 1 mL of nutrient medium containing RPMI-1640 (Sigma-Aldrich, St. Louis, MO, USA), 10% inactivated (for 30 min at 56 °C) fetal bovine serum (Sigma-Aldrich, St. Louis, MO, USA), 50 mg/L gentamicin (Invitrogen, Glasgow, UK) and freshly added L-glutamine sterile solution in a final concentration of 0.3 mg/mL (Sigma-Aldrich, St. Louis, MO, USA). Iron oxide maximum tolerated dose (MTD) was calculated on the basis of iron ions: one MTD of iron in water was equal to 0.3 mg/L. Then FF1 or FF2 suspensions of magnetic nanoparticles were immediately added to cell culture in final concentrations of 2.3, 10, 100 or 1000 MTDs. Both suspensions were pipetted fivetimes. The control cell culture (0 MTD) had nether MNPs nor chitosan solution. The cell cultures were incubated for 24 h in atmosphere of 95% air and 5% CO_2_ at 37 °C. Preliminary study showed no sign of chitosan toxic effect on HBMLs culture. After incubation, the cell suspension was centrifuged at 1300 rpm for 10 min. The obtained cell pellet was used to measure cell death, membrane antigen presentation, transmission electron microscopy (TEM), and energy dispersive X-ray fluorescence analysis. The cell culture supernatants were employed to measure cytokine concentrations. 

### 2.4. Measurement of Cell Death

Calculations of cell concentration and viability were conducted with a Countess TM Automated Cell Counter (Invitrogen, Carlsbad, CA, USA) using 0.4% trypan blue solution (Invitrogen, Carlsbad, CA, USA). To this end, the cells to be tested were transferred in a volume of 12.5 μL to an immunological plate, and 12.5 μL of stain was added. Viable cells were unstained by 0.4% trypan blue.

### 2.5. Cytokine Profile in the Cell Culture

Cytokines are small peptide information molecules. Cytokines have a molecular mass not exceeding 30 kD. Their main producers are lymphocytes. Cytokines regulate intercellular and intersystem interactions, determine cell survival, stimulation or suppression of their growth, differentiation, functional activity and apoptosis, and also ensure the compatibility of the action of the immune, endocrine and nervous systems under normal conditions and in response to pathological effects. Cytokines are active in very low concentrations. Their biological effect on cells is realized through interaction with a specific receptor located on the cell cytoplasmic membrane. The formation and secretion of cytokines occurs briefly and it is strictly regulated. In the present work, to measure the spontaneous secretion of interleukins (IL-2, IL-4, IL-6, IL-8, and IL-10),granulocyte-macrophage colony-stimulating factor (GM-CSF), interferon-gamma (IFN_γ_), and tumor necrosis factor alpha (TNF_α_) in the supernatants (intracelular fluids), a flow cytometry (FC) was performed. The FC procedure was conducted according to the instructions of the manufacturer of the cytokine assay system (Bio-Plex Pro Human Cytokine 8-Plex Panel, Bio-Rad, Hercules, CA, USA) using an automated processing system (Bio-Plex Protein Assay System, Bio-Rad, USA). The concentration of each cytokine was expressed in pg/mL. 

### 2.6. Cellular Immunophenotype Detection

The cellular antigen profile was analyzed using a method based on the interaction between specific monoclonal antibodies (mAbs; see below) and clustering determinants on the cell surface according to the manufacturer’s instructions. After culturing, the cells were washed with phosphate-buffered saline (рН 7.2), and a single-cell suspension in a volume of 10 μL was mixed 1:1 with a single standard mAb against CD45, CD3, CD4, CD8, CD25 (Abcam, Cambridge, UK), CD28, CD45RO, CD45RA, CD71, or CD95 (e-Bioscience, San Diego, CA, USA). The mAbs were labeled with fluorescein isothiocyanate (FITC), allophycocyanin (АРС), phycoerythrin (PE), or peridinin chlorophyll protein (PerCP). After 30 min of incubation with the labeled mAb, the cells were assayed using a MACS Quant flow cytometer (Miltenyi Biotec, Teterow, Germany). Measurements of the orange/green/red fluorescence parameters were performed at the gate of the analyzed cells, and the numbers of cells presenting the studied antigenic determinants were calculated. The cytometric results were examined using KALUZA Analysis Software (Beckman Coulter, Indianapolis, IN, USA).

### 2.7. Electrochemical Testing

Concentrations of iron oxide MNPs in biological liquids were confirmed by stripping voltammetry (SV) of iron ions [25].Calculated maximum tolerated doses in medium were compared to those estimated by SVas described in [12].

### 2.8. Transmission Electron Microscopy of Cells

The protocol to visualize cells under TEM was follows. Cell culture was washed in PBS at 37 °C and pre-fixed in 10 mL of 0.5% glutaraldehyde in Sörenson buffer at room temperature for 15 min. After pre-fixation, cells were decanted into a 15 mLFalcon tube and centrifuged at 1500 rpm for 10 min. As the next step the supernatant was removed and freshly made 2% glutaraldehyde in Sörenson buffer added to the pellet. After 2 h fixation the pellet was spun at 2000 g for further compaction. Samples were fixed in 1% Osmium Tetroxide in Sörenson buffer, dehydrated, embedded in Epon Polarbed resin and cut as 70 nm ultrathin sections for TEM studies (EM208S, Philips, Moosseedorf, Germany).

To compare iron oxide MNPs distribution in different cells the primary culture of post-natal adipose-derived multipotent mesenchymal stromal cells (AMMSCs) was prepared from human fate tissue after processing of lipoaspirates (Permission No. 4 from 23.10.2013 of Local Ethics Committee of Innovation park of Immanuel Kant Baltic Federal University). AMMSCs culturing and TEM was prepared as described in our previous work [12].

### 2.9. Statistical Analysis

The results were analyzed using STATISTICA software for Windows 10.0. The following distribution parameters were calculated: the median (Me), the 25% quartile (Q1) and the 75% quartile (Q3). The Mann-Whitney U-test (P_U_) was performed, and differences were considered significant at *p* < 0.05. The relationship between the studied parameters was established via regression analyses. The coefficients (r) were kept at a significance level greater than 95%.

### 2.10. Energy Dispersive X-ray Fluorescence Analysis of Cells

Total reflection X-ray fluorescent spectrometry (TXRF) [26] is a relatively new method of elemental analysis that can be applied to samples in the form of thin layer including dried drops of homogenized suspensions of fine particles or a thin layer of whole cells. Although the TXRF method without a pressure controlled chamber proved to be limited for low Z elements quantification (such as carbon, nitrogen, and oxygen) [27,28], it can be successfully applied for the determination of elements with higher-energy characteristic lines. All TXRF measurements were carried out by a Nanohunter spectrometer (Rigaku, Tokyo, Japan). For every experiment the same parameters were used: exposure time of 500 s, angle of 0.05°, X-ray tube withCu anode as a primary beam source. Samples were dried at 50 °C in a Rigaku Ultra dry chamber at normal pressure. For all calculations of iron concentration, we neglected the matrix effects assuming thin film sample geometry.

## 3. Results and Discussion

**Figure 2:**
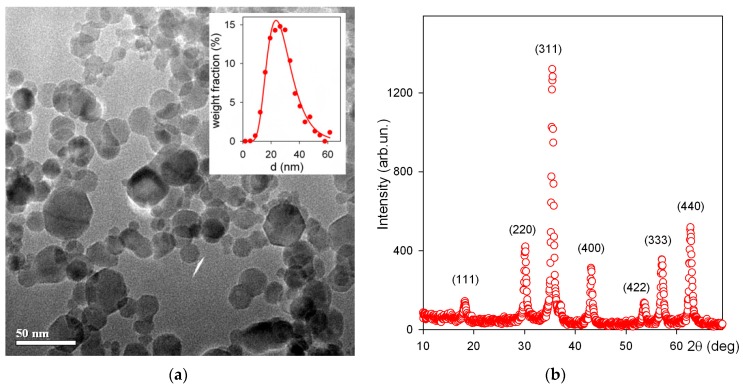
TEM image of iron oxide MNPs (JEOL JEM2100 operating at 200 kV). Inset: particle size distribution (number averaged) obtained by the graphical analysis of TEM images (2160 particles) (**a**). XRD plot for iron oxide MNPs (Bruker D8 DISCOVER) Cu-K_α_ radiation (λ = 1.5418 Å), a graphite monochromator and a scintillation detector (**b**).

Figure 2a shows a typical TEM microphotograph of iron oxide MNPs synthesized by the LTE technique. The MNPs were non-agglomerated and their shape was close to spherical. Only few of the particles appeared to be hexagonal or having hexagonal corners. Weighted particle size distribution (PSD) (Figure 2a inset) was lognormal, with a median value of 26.8 nm and dispersion 0.362. The specific surface area of MNPs was 78 m^2^/g. The surface average diameter of MNPs, calculated from this value using the equation d_s_ = 6/(ρ × S_sp_) (ρ = 4.6 g/cm^3^ being iron oxide density) was 16.7 nm. It was in good agreement with the value d_s_ = 15.9 nm, obtained using PSD with aforementioned parameters. XRD plot of iron oxide MNPs is given in Figure 2b. 

The crystalline structure of MNPs corresponded to the inverse spinel lattice with a space group Fd3m. The lattice period (a) was found a = 0.8358 nm, which was larger than that for iron oxide (γ-Fe_2_O_3_, a = 0.8346 nm) but lower than that for magnetite (Fe_3_O_4_, a = 0.8396 nm) [29]. Based on the dependence between the lattice period of the spinel cell and the effective state of oxidation of Fe the composition of MNPs was defined. It contained 76% of γ-Fe_2_O_3_ and 24% of Fe_3_O_4_.

Average hydrodynamic diameters of aggregates in suspensions were monitored by the dynamic light scattering (Brookhaven Zeta Plus). The concentration of ferrofluid after centrifuging was 5.0% of MNPs by weight for FF1 suspension. The average hydrodynamic diameter of MNPs in suspension was 56 nm. 

**Figure 3:**
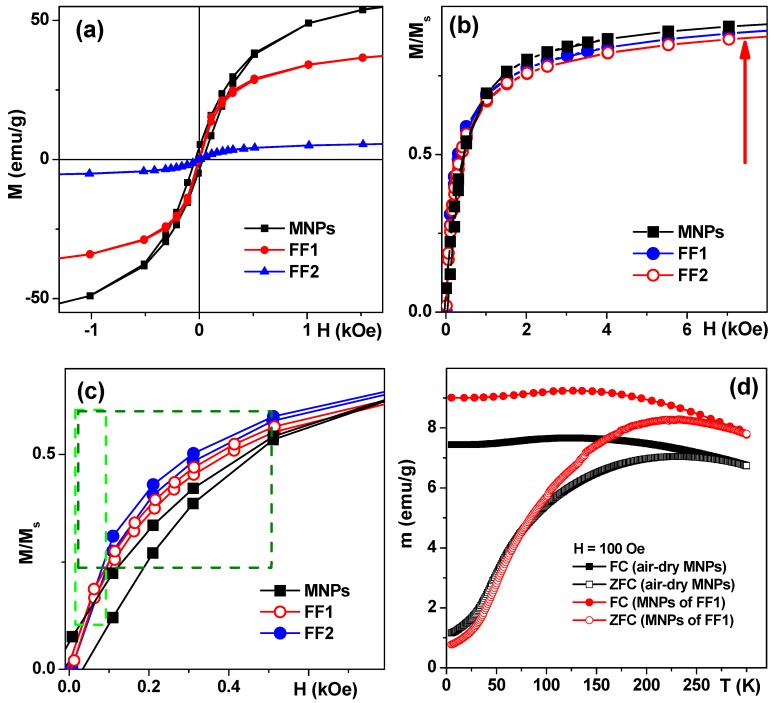
(**a**)—Hysteresis loops of LTE MNPs, FF-1, FF-2 suspensions at room temperature. Recalculated M/M_s_ hysteresis loops for M_s_—being a saturation magnetization values corresponding to each particular case (**b**,**c**), red arrow shows the direction of the increase of the magnetization; (**c**)—dashed lines show two important intervals for possible applications: light green—magnetic biosensing interval; green—cell use as native microcapsules for targeted delivery in a supplement of nanomedicine, theranostics, cell technologies, and regenerative medicine; (**d**)—ZFC-FC curves for air dry LTE MNPs and MNPs of FF1.

Magnetic measurements confirmed that MNPs were close to a superparamagnetic state with low coercivity (H_c_) at 20 °C (Figure 3a). Following H_c_ values were obtained for air-dried MNPs—30 Oe, FF1 MNPs—5 Oe and FF2 MNPs—5 Oe. It is noteworthy that the saturation was not totally reached at a maximum available field of 70 kOe. The saturation magnetization (M_s_) value of about 70 emu/g for MNPs is in good agreement with about 20 nm sized MNPs of maghemite [22,30]. The coercivity decrease down to about 5 Oe and Ms decrease for FF1 and FF2 MNPs were consistent with the separation process during the suspension fabrication and the fact of the reduction of the mass of magnetic material per unit mass due to sodium citrate and chitosan incorporation. Although analysis of the transition between SPM behavior and blocked state was not the subject of the presents study in order to demonstrate very roughly the features of magnetic interactions we show the results of ZFC-FC measurements for air-dry LTE MNPs and MNPs obtained by drying the FF1 ferrofluid. For used concentrations MNPs are supposed to be weakly interacting but the ensemble can contain coarse particles which contribute much to coercivity and complex shape of ZFC–FC thermomagnetic curves (Figure 3d). Even so these curves additionally verify the MNP superparamagnetic behavior in the ensemble under study. Thus, the blocking temperature of about 150 K typical for superparamagnetics can be seen. It is interesting that the parameters of ZFC–FC thermomagnetic curves for MNPs produced from dried aqueous suspension FF1, are very close to those for air—dry MNPs. The latter is explained by a small original size of the MNPs, i.e., their separation during the preparation of magnetic suspensions has no considerable effect on the size distribution parameters.

**Figure 4:**
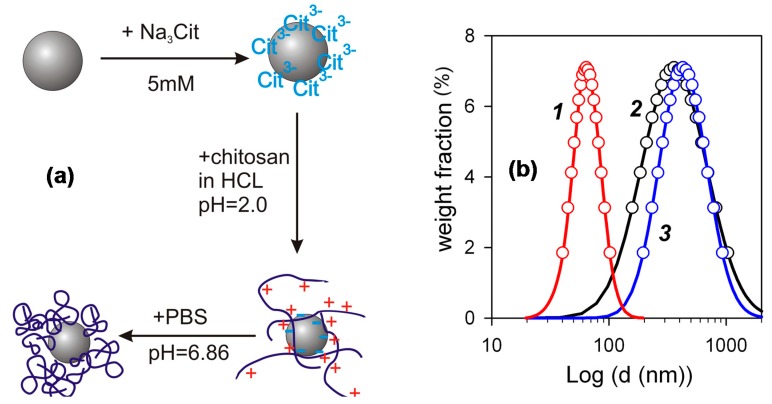
(**a**)—Scheme of encapsulation of iron oxide MNPs by chitosan. (**b**)—PSD (obtained by DLS) in suspensions at different steps of the encapsulation: 1—suspension FF1 of MNPs in 5 mM citrate—solution of chitosan taken for encapsulation—suspension FF2 of encapsulated MNPs.

The FF2 suspensions of iron oxide MNPs for biocompatibility studies were prepared through several steps schematically shown in Figure 4a. First, a water suspension of individual MNPs electrostatically stabilized by adsorbed surface layer of citrate anions was prepared. Its zeta-potential was –48 mV, which provided efficient mutual repellence of negatively charged MNPs and prevented their aggregation. The next step was the treatment of MNPs with the acidic solution of chitosan, which resulted in the adsorption of chitosan polycations on the negatively charged surface of MNPs. The adsorption of chitosan was not, however, purely electrostatic, because it resulted in the inversion of the surface charge of the MNPs. Their zeta-potential after the adsorption step was +52 mV, which was very close to the zeta-potential of the chitosan solution (+50 mV). It means that the total positive charge of adsorbed chitosan exceeds the negative charge located at the surface of MNPs. Most likely the bonding of chitosan macromolecules to the surface stems from the molecular interactions due to amine and hydroxyl groups in monomer units (see Figure 1). The final step of preparation was the adjustment of pH to neutral level by PBS, which caused the de-ionization of chitosan and the contraction of its coils around the MNPs. The details and the physicochemical background of such an encapsulation of iron oxide MNPs by chitosan was given in our previous studies [19,20].

Figure 4b shows PSD (obtained by DLS) of species in the suspension of iron oxide MNPs ecapsulated by chitosan in comparison with PSD in the precursor suspension of MNPs stabilized by citrate and with PSD in the solution of chitosan. One can see, that in the case of PSD of the suspension FF1 the median value of the diameter was about 50 nm. It is higher than the weight average value of the diameter determined in the graphical analysis of TEM images (about 30 nm). The difference, however, is not large and may likely be attributed to the thickness of solvating shells and the thickness of the double electric layer at the surface of the particles, which provide the stability of their suspension. The median diameter of PSD for the solution of chitosan and for the suspension of the encapsulated MNPs is much larger. In both cases it is around 300 nm. The obtained value for the chitosan solution is much higher than that anticipated based on the molecular parameters of chitosan.

The characteristic dimension of the polymeric chain in the solution is the mean square end-to-end distance for the macromolecular coil, which can be estimated using the following equation [31]:

(1) $$\langle R^{2}\rangle =NA^{2}$$

where *N* is the number of statistical segments in the macromolecule and *A* is the length of the segment.

The length of the segment for chitosan varies in different reports [32], instead the value 10 nm might be taken as a fair estimation. It corresponds to ca. 20 monomer units in the segment. The average total number of monomer units in chitosan used in the study can easily be evaluated as a ratio of the molecular weight of chitosan to the formula molecular weight of the unit (148). Thisgives ca. 3000 units in chitosan and, hence, ca. 150 segments in the macromolecule. The calculation of end-to-end distance based on these values gives ca. 150 nm, which is two times lower than the median diameter of PSD for chitosan solution. It means that the solution of chitosan does not contain individual macromolecules but their aggregates. This result is consistent with published reports on the association and aggregation in chitosan solution [33,34]. According to them association is inevitable and occurs even in filtered dilute solutions being kept for some period of time in isothermal conditions. We had obtained the mean value of the diameter of 80 nm for the acidic solutions of chitosan with 0.002 g/L concentration filtered through Wattman 0.1 μm filter. Meanwhile, the associates were re-established after the three-day period of storage of the solution at 25 °C. It is obvious from Figure 4 that the diameter of encapsulated MNPs is the same as the diameter of chitosan aggregates. It means that the encapsulation of individual MNPs by chitosan can possibly be achieved only in very dilute solution; but in the solutions of finite concentration the process of interaction among chitosan and MNPs rather ends up in the incorporation of MNPs into the associates (aggregates) of chitosan macromolecules. 

**Figure 5:**
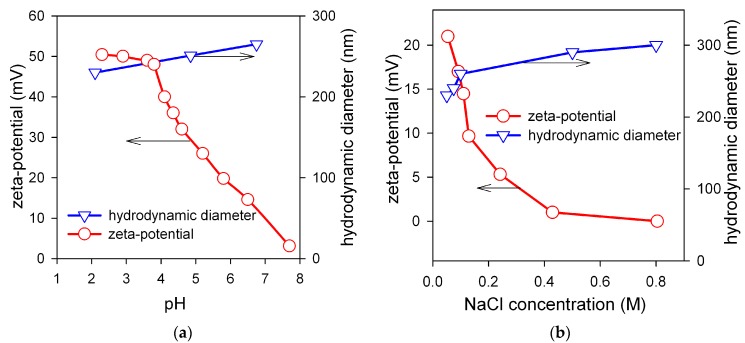
(**a**)—pH dependence of the zeta-potential and of the mean hydrodynamic diameter of aggregates in the suspension FF2 of the encapsulated iron oxide MNPs. (**b**)—Dependence of the zeta-potential and of the mean hydrodynamic diameter of aggregates in the FF2 suspension of the encapsulated MNPs at pH = 6.5.

Figure 5a shows the influence of pH on the zeta-potential and on the mean hydrodynamic diameter of aggregates in the suspension of the encapsulated MNPs. Zeta-potential is almost constant in the pH range up to pH = 4 and gradually diminishes with the further increase of pH. It reaches values below +20 mV in the physiologically relevant range of pH = 6.5–7.5. The decrease of zeta-potential happens due to the deprotonation of amine groups in the chitosan macromolecule according to the reaction:

Chit–NH_3_^+^Cl^−^ + OH^−^ => Chit–NH_2_ + H_2_O + Cl^−^

As the positive electric charge of chitosan macromolecules decreases, the electrostatic factor of stability of the suspension diminishes. In principle, it is unfavorable to the stability, as it is known from the theoretical consideration in terms of the Derjaguin-Landau-Verwey-Overbeek (DLVO) approach that the zeta-potential should maintain the value above 30 mV irrespective of the sign [35] to provide the stability of the suspension against the aggregation due to the attractive Van-der-Waals forces between MNPs. Meanwhile, the suspension of encapsulated MNPs retains its stability, and the mean hydrodynamic diameter of chitosan associates does not increase substantially. This effect is totally due to the steric repulsion among chitosan aggregates with embedded iron oxide MNPs. This repulsion stems from the osmotic forces, which appear if the polymeric shells of aggregates overlap. In this case the local concentration of segments increases in the overlapping region and it causes the incoming osmotic flux of water, which prevents interpenetration of aggregates and keeps them apart. 

Figure 5b shows the influence of salt (NaCl) concentration on the zeta-potential and on the mean hydrodynamic diameter of aggregates in the suspension of the encapsulated MNPs at pH = 6.5. The zeta-potential of the suspension decreases substantially if salt is added due to the contraction of the double electric layer on the surface of chitosan aggregates. At NaCl concentration 0.4 M zeta-potential vanishes to zero. It means that the electrostatic factor of the stability of the suspension is effectively eliminated by the ionic force of the salt. Meanwhile, the level of aggregation indicated by mean hydrodynamic diameter of chitosan associates retains almost the same. Certainly, it is the result of the efficient steric stabilization of the encapsulated MNPs in FF2 suspension. 

In general, the dependences given in Figure 5 show that the encapsulation of LTE iron oxide MNPs by chitosan makes them resistant to unfavorable factors for the colloidal stability such as pH and high ionic force, which are typically present under physiological conditions. It makes the suspensions of MNPs encapsulated by chitosan promising candidates for the biocompatibility testing. Therefore, as the next step biological experiments were provided for comparative analysis of the biocompatibility of the FF1 and FF2 suspensions for the case of human blood mononuclear leukocytes.

According to the experimental data, intact HBMLs express a wide range of membrane markers in a 24-h in vitro culture. 99% of the cells express CD45CD3 T-lymphocyte antigens, predominantly (55%) of CD45RA^+^ naive (antigen non-activated) CD4^+^ T-helper/inducers (67%) [36,37]. Membrane activation and co-stimulatory molecules CD25, CD28, CD71 and CD95 are present for CD45CD3^+^ cells in 9%; 79%; 2.5% and 15% of cases, respectively. CD45RO isoform of the transmembrane antigen expressed in vitro on activated T-lymphocytes and/or T-memory cells was detected on 35% for CD45CD3^+^ cells [37,38] (Table 1).

24-h culture of the viable HBMLs (median of living cells was 93.5%, Table 2) spontaneously secreted into the intercellular fluid the spectrum of immunomodulating cytokines and chemokines with pro-inflammatory (IL-2, IL-8, TNFα, GM-CSF, IFNγ) and anti-inflammatory activity (IL-4, IL-10) [39] capable of modulating the in vitrosurvival, proliferation, differentiation and maturation of HBMLs through autocrine/paracrine signaling pathways. It should be emphasized specially that both tested suspensions (without and with chitozan) did not cause cell death in 24-h culture in vitro (Table 2), which implies a non-toxic but irritating effect of massive doses of MNPs on the secretory function of HBMLs.

In the 2.3–1000 maximum tolerated dose (MTD) range, FF1 suspensions (without chitosan) produced a dose-dependent statistically significant increase (2–47 times with respect to the baseline) of the secretion of all cytokines studied (Table 3). Significantly, the concentration of IL-10 in the intercellular fluid increased in an exponential dependence on the dose of MNPs without chitosan (FF1 suspension) (Figure 5). 

We observed the most indicative difference in dynamics of changes in suspensions of MNPs with and without chitosan in the range of 10–1000 MTD for the case of IL-10. With chitosan, a decrease in cytokine secretion in this range of low doses very pronounced. At the same time, the relative increase in the secretory activity of HBMLs in the range of 100–1000 MTDs was slowed down with the dependence on the plateau of the classical S-shaped dose-response curve (Figure 5 and Table 3).

Dispersing LTE iron oxide MNPs in a chitosan matrix changed the type of the dose-response curve for the secretion of biological molecules by HBMLs culture. Figure 5 shows the summary of these results: curve 1 (corresponding to the FF1 suspension without chitosan) was well fitted by exponent *y* = 2.13e^1.06*x*^, R^2^ = 0.96; curve 2 (corresponding to the FF2 suspension with chitosan) was well fitted by 3-degree polynomial dependence with a high degree of approximation: *y* = 6.5*x*^3^ + 51.9*x*^2^ + 124.1*x* − 73, R^2^ = 0.99 and curve 3 showing correlation between calculated dependence of iron concentration in MTDs and in parts per million (ppm) was well fitted by exponent *y* = 0.0119e^1.9785*x*^, R^2^ = 0.99, wherein, the value of 2.3 MTDs of MNPs with chitosan statistically increased the concentrations of all tested cytokines, compared to both levels—the baseline level and the corresponding dose of MNPs without chitosan (Table 3). 

On the contrary, in the range of 10 to 100 MTDs of MNPs with chitosan leveled the stimulating effect of MNPs on the secretory activity of HBMLs. At 100 MTDs, a decrease in the concentrations of IL-2, IL-4, IL-6, IL-10 cytokines reached the baseline level. At 1000 MTDs, the failure of secretion caused by MNPs plus chitosan was replaced by an overshoot (Table 3), which in only two cases (IL-8, GM-CSF) significantly exceeded the corresponding values reached with the suspension FF1 without chitosan. The obtained data made it possible to formulate a working hypothesis:

(1) on the uneven distribution of different doses of MNPs in the chitosan matrix;
(2) the predominantly retarding effect of chitosan on the irritating effect of high (10–1000 MTDs) doses of magnetic maghemite nanoparticles.

At that, 100 MTDs turned out to be a critical (reference) point for research. To test the hypothesis, additional experiments with cell culture (TEM of biological samples, energy dispersive X-ray fluorescence analysis) were carried out. Calculated MTDs of MNPs in the medium corresponded to those estimated by stripping voltammetry. Thus, the electrochemical analysis of supernatants providing for the complete dissolution of MNPs during sample preparation showed the result of three measurements of the concentration of iron 33 ± 11 mg/kg at 100 MTDs and 2.9 ± 0.9 mg/kg at 10 MTDs. It is to be recalled that the maximum tolerated dose (MTD) is 0.3 mg/L iron ions in water solution. 

**Figure 6:**
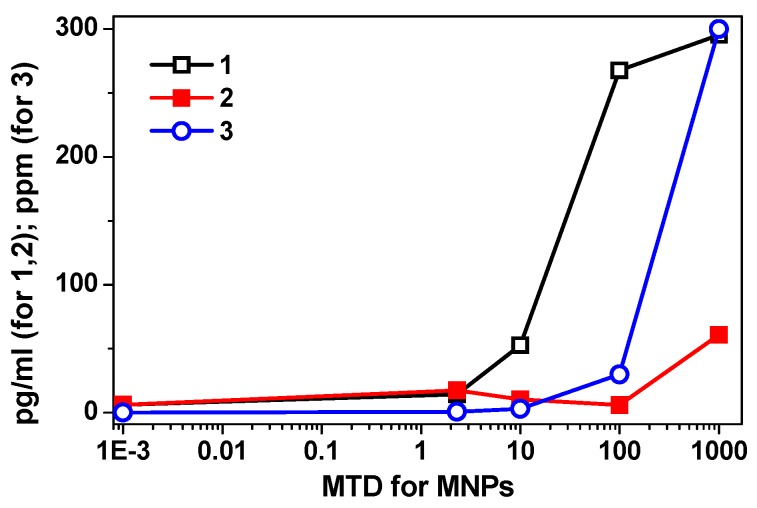
An example of modulating effect of suspensions on cytokine (here, IL-10), concentrations are given for 24-h HBMLs culture. Curve 1 corresponds to the FF1 suspension without chitosan, curve 2 corresponds to the FF2 suspension with chitosan and curve 3 shows correlation between calculated dependence of iron concentration in MTDs in ppm.

Energy distribution X-ray fluorescence analysis of cells and supernatants showed (Table 4) the uneven distribution of MNPs in the cell fraction and intercellular fluids (supernatants) in low-speed centrifugation of HBMLs. According to Table 4, the measured iron concentration in the suspension of MNPs without chitosan at a maximum of 1000 MTDs was significantly lower than the calculated one presented in Figure 6. In contrast, the measured concentration of MNPs with chitosan at 100 MTDs (Table 4), primarily in the cell fraction, was significantly (3–10 times) higher than the calculated (Figure 6 and Table 4). On one hand, we should make a special remark related to the low solubility of iron oxide nanoparticles in the time being tested (24 h) [40] and therefore the existing ambiguity in determining the MTDs in the case of MNPs. 

Strictly speaking the maximum tolerated dose definition is given for the ionic form of iron: 0.3 mg/L of Fe ions in water. Nanoparticles and, in a broader sense, colloidal particles, strictly speaking, cannot be considered a soluble form. The particles are a separate insoluble phase with an interface with the aqueous medium. Although some iron solubility on the boundary of the particle is possible, but most likely it does not exceed value around one MTD. The observed high error in the iron concentration definition by TXRF analysis (30–300 ppm) may be a consequence of quite non-uniform iron distribution in the biosample when non-dissolved composites were measured. With the low solubility of iron oxide nanoparticles in the time being tested, probably an instrumental approach to determination of high doses of MNPs similar to soluble forms of iron is not entirely applicable. On the other hand, chitosan seems to have contributed to the accumulation of MNPs in the cell fraction at high iron concentrations after centrifugation as compared with supernatants (Table 4). Therefore, TEM studies of HBMLs fractions incorporated into resin were studied in order to determine the possible localization of nanoparticles with respect to cells at 100 MTDs grows conditions.

**Figure 7:**
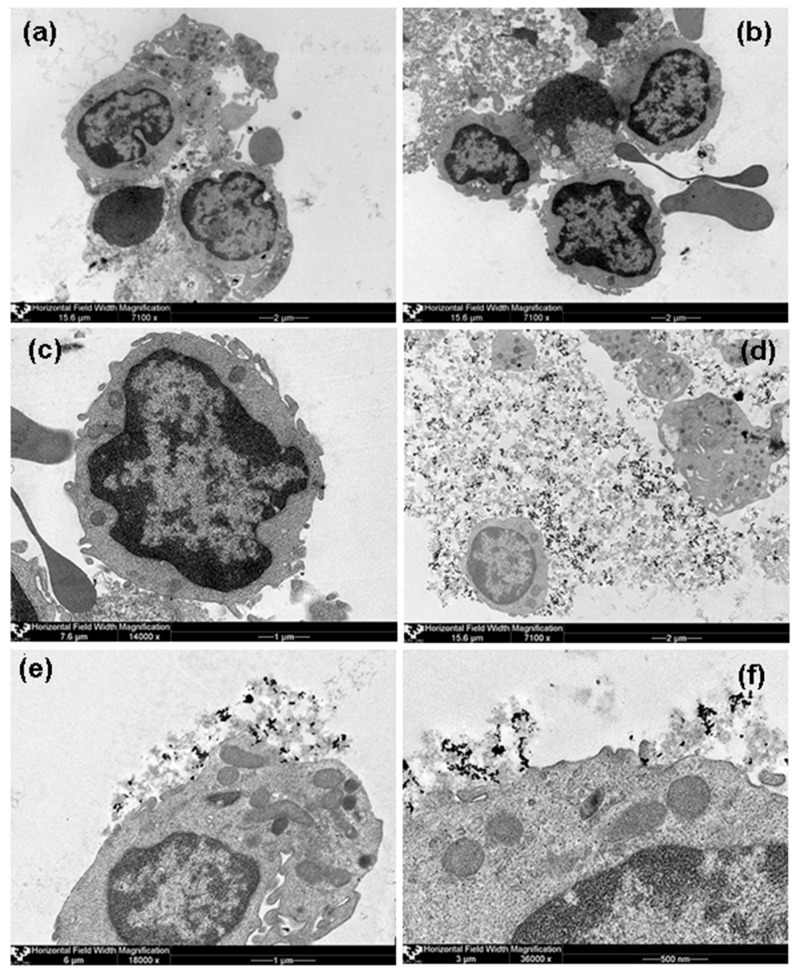
TEM of HBMLs mononuclear blood leukocytes. (**a**)—control culture grown without MNPs. HBMLs mononuclear blood leukocytes after 24-h contact with MNPs of suspension of iron oxide MNPs without chitosan (FF1) at a dose of 100 MTDs: (**b**)—magnification ×7100, (**c**)—magnification ×14,000. HBMLs after 24-h contact with MNPs of suspension of iron oxide MNPs with chitosan (FF2) at a dose of 100 MTDs, magnifications: (**d**)—×7100, (**e**)—×18000, (**f**)—×36000.

Analysis of TEM images showed rather dispersed intracellular distribution of individual MNPs in HBMLs cellswithout the formation of conglomerates in both tested cases (with and without chitosan). Figure 7 demonstrates some representative examples, including general view of control culture grown without suspension of MNPs but in the same conditions. Due to the visually small penetration of MNPs into the HBMLs, we failed to make a comparative quantitative assessment of their intracellular concentrations. In addition, in the case of MNPs without chitosan (FF1), nanoparticle agglomerates were not detected in the intercellular environment (supernatants), that is individual MNPs were dispersed outside the cells, and only very few on the cytoplasmic membrane and inside the cells. At the same time, MNPs with chitosan (FF2) outside the cells were concentrated in the form of agglomerates in the chitosan matrix fixed as separate “flakes” to the outside of the cytoplasmic membrane of HBMLs. Apparently, chitosan prevented the free penetration of MNPs into HBMLs. On the other hand, it caused uneven distribution of MNPs, which contributed to an excess of the calculated dose of 100 MTDs when non-dissolved composites were measured. 

**Figure 8:**
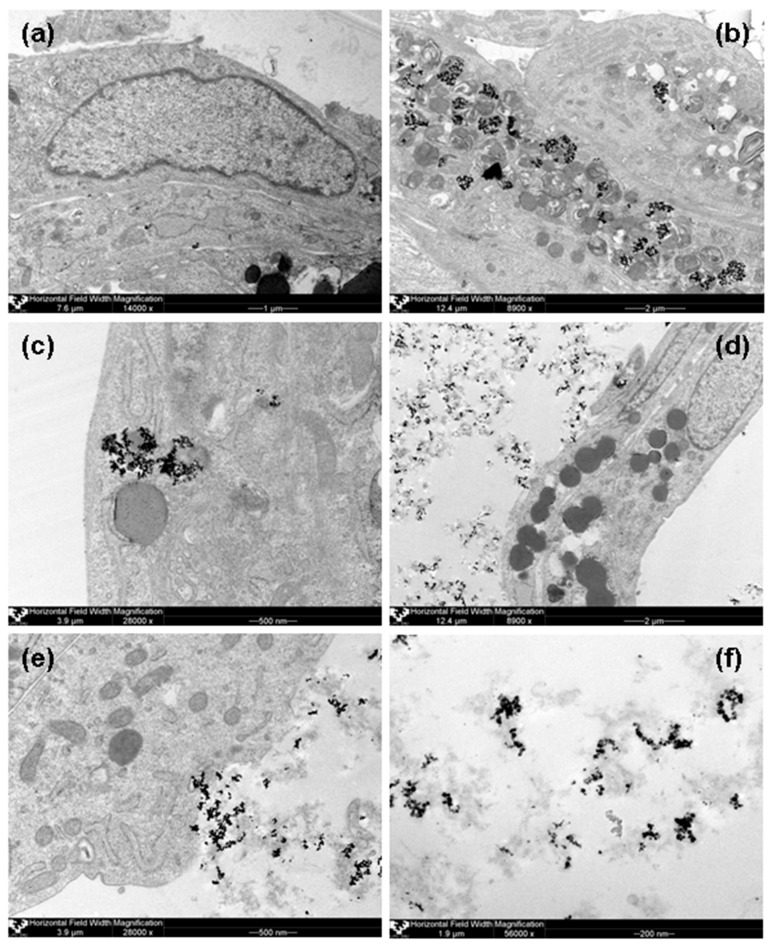
TEM of human mesenchymal stem cells. (**a**)—control AMMSCs culture grown without MNPs. AMMSCs cells after 24-h contact with MNPs of suspension of iron oxide MNPs without chitosan (FF1) at a dose of 100 MTDs: (**b**)—magnification ×8900, (**c**)—magnification ×28,000. AMMSCs cells after 24-h contact with iron oxide MNPs of suspension with chitosan (FF2) at a dose of 100 MTDs: (**d**)—magnification ×8900, (**e**)—magnification ×28,000. Aggregates of LTE MNPs encapsulated by chitozan (**f**)—magnification ×56,000.

Recently we have performedin vitro experiments with human adipose-derived mesenchymal stem cells (AMMSCs) underthe same conditions: stable colloidal suspensions using electrostatic or steric (by chitosan) stabilization of iron oxide MNPs obtained by LTE for 100 MTD MNPs concentration and after 24 h contact with suspension [12]. Intracellular MNPs inclusions were clearly observed in mainly inside organelles of AMMSCs contacted with 100 MTD of FF1. MNPs aggregates were noted inside endosomes, contoured the hydrolytic vesicles/secretory granules and outer membrane of mitochondria and only single inclusions were freely situated in cellular cytoplasm. Mitochondria looked like myelin-like bodies with hyperelectron-dense inclusions between membranous layers. In the case of FF2 suspension hyperelectron-dense aggregated MNPs They seemed to enter into the cells to a lesser extent in comparison with FF-1 MNPs: very few inclusions contoured only the hydrolytic vesicles/secretory granules. For sake of comparison Figure 8 shows AMMSCs images for the same conditions as those used for the Figure 7 cases.

Figure 8f shows very interesting case—MNPs of FF2 suspension as the aggregates in chitozan matrix. Very roughly one can estimate the average size of the aggregate to be the order of 50 to 200 nm and containing approximately 20 to 100 nanoparticles. Aggregates have large variety of shapes but they do not tend to be very spherical but rather characterized by the presence of various chains of different length. Interestingly, the average size of the aggregates observed by TEM was close to the calculated of end-to-end distance for the segment for chitosan (150 nm), which is two times lower than the median diameter of PSD for chitosan solution. In the case of the MNPs aggregates inside the AMMSCs, the average size of the aggregate concept is not applicable as the size of the MNPs aggregates predetermined by the size of the cell organelles. In both cases (HBMLs and AMMSCs) the size of the main organelles like mitochondria was much larger comparing with the size of typical MNPs aggregates for FF2.

It is important to mention that T-lymphocytes (CD45+CD3+ cells) account more than for 95% of all mononuclear cells (Table 1). The difference between T-lymphocytes and the AMMSCs is that T-lymphocytes are not phagocytic cells. Previously we observed that AMMSCs can accumulate tested MNPs in endosomes, secretory granules and mitochondria (Figure 8). As a consequence, our suppositions based on the obtained data were: (1) the existence of uneven distribution of MNPs in chitosan due to agglomeration of the latter with cell membranes, (2) the existence of the predominantly inhibitory effect of chitosan for the irritating effect of high (10–1000) MTDs of magnetic maghemite nanoparticles on cytokine secretion. Perhaps the observed phenomenon is caused by a decrease in the penetration of MNPs into cells and enveloping cells with chitosan flakes, which can lead to a decrease in the free secretory cell surface or the fixation of cytokines in chitosan matrix. “Enveloping” effect is an interesting phenomenon, because usually the main attention is attracted to the phenomenon of the irritating effect of the nanoparticles. In practical terms, this may find application in the future when developing biotechnological methods restriction of complications of cellular allergic reactions similar to hypersensitivity of delayed type, for example, with bronchial asthma (as a spray) or dermatitis. The question of why this effect manifests itself, mainly at 100 MTDs, remains open and extremely interesting for interdisciplinary research. It is possible that maghemite MNPs passively does not enter the cells due to special combination of the factors between others the zeta potential and magnetic interactions may play important roles.

As we have confirmed the possibility of either massive incorporation of LTE MNPs into AMMSCs (FF1) or their adhesion toward the cell membrane (HBMLs, AMMSCs for FF2) one can discuss the possibility of their magnetic filed assisted application. To make the comparison easier let us re-calculate M/M_s_ hysteresis loops for Ms—being a saturation magnetization for each particular case (Figure 3). Figure 3a shows that the saturation magnetization of air-dry MNPs is the highest one and M_s_ for FF2 MNPs is the lowest as one could expect taking into account the fabrication process and inevitable separation step resulted in the removal of large MNPs. At the same time in low field interval below 1 kOe, which is most reasonable for applications, one can observe that FF1 and FF2 MNPs reach the higher moment comparing with air-dry in the same low field. This is clear advantage for such application as magnetic biosensing (magnetic fields below 100 Oe [12]). The second interval for possible applications (100–500 Oe) is connected to idea of the cell use as native microcapsules for targeted delivery in a supplement of nanomedicine and theranostics, cell technologies, and regenerative medicine [41,42,43].

Recently we have shown that the presence of LTE MNPs changes the physical properties of ferrogels synthesized by radical polymerization of acrylamide and their biocompatibility [43]. We found that the gradual increase of MNPs concentration in the gel network resulted in the significant increase of the negative value of electrical potential and adhesion index for both the human dermal fibroblasts and the human peripheral blood leucocytes. Ferrimagnetic MNPs affect hemopoietic and stromal cells and promote cellular adhesion and formation of cell-to-cell contacts along the magnetic field lines. Thisimplies cell use as native microcapsules for targeted delivery in a supplement of nanomedicine and theranostics, cell technologies, and regenerative medicine. From viewpoint of biomedical applications, the inclusion of small amount of LTE MNPs into the polymer network seems to be very positive step which significantly enhances the mechanical and electrical properties of ferrogels, and improves biocompatibility of these systems. Although, this point was not yet investigated, one can expect certain influence of the applied magnetic field on the adhesion process. Here we can propose to plan the experiments with LTE MNPs loaded cells (AMMSCs cells after 24-contact with MNPs of suspension of iron oxide MNPs without chitosan (FF1) or of HBMLs mononuclear blood leukocytes. after 24-contact with LTE MNPs of suspension of iron oxide MNPs with chitosan (FF2) at a dose of 100 MTDs). 

Even more sophisticated applications can be thought. The majority of the present day in vitro studies are focused on the understanding of one particular culture. In the case of HBMLs and MMSC cells studied in similar conditions we found clear difference in MNPs internacionalization process for FF1 case. This means that LTE MNPs will affect specifically on HBMLs if both cells are exposed together, the situation which seems to be realistic from practical point of view. 

One of therapeutical problems of MNPs applications is rapid recognition of MNPs by the immune system [44]. LTE MNPs encapsulation by MMSC cells can be a good solution for certain stage of the treatment especially taking into account the possibility to manipulate living cells anchored with MNPs by a magnetic field. As for therapeutically purposes like hyperthermia or thermal ablation the excess of MNPs is always necessary there is a request for new techniques of removal of the excess of MNPs after the therapy. One can propose low invasive cathetering of ferrogel pads with adhered cell culture toward the region of the MNPs excess (which may be concentrated by the external magnetic field application) after the pad cells loading with MNPs pad can be removed providing removal of the MNPs excess. Of course, these hypothetic scenarios require special long series of investigation steps but the general directions seems to be promising.

## 4. Conclusions and Outlook

Magnetic iron oxide nanoparticles were obtained by a highly productive laser target evaporation technique. XRD analysis confirmed their inverse spinel structure (space group Fd-3m). According to TEM, the shape of MNPs was close to spherical. The analysis of TEM images revealed that the ensemble obeyed lognormal size distribution with amedian value of 26.8 nm and 0.362 dispersion. Stabilized water-based suspensions with MNPs were fabricated using electrostatical or sterical stabilization by chitosan. It was shown that the encapsulation of iron oxide MNPs by chitosan makes them resistant to unfavorable factors for the colloidal stability such as pH and high ionic force, which are typically present under physiological conditions. This makes the suspensions of MNPs encapsulated by chitosan promising candidates for biocompatibility testing.

Controlled amounts of suspensions were used for in vitro experiments with human blood mononuclear leukocytes in order to study their morphofunctional response in the wide Fe concentration range. Special efforts were made in order to provide complete physical and physical-chemical characterization of obtained MNPs and suspensions. For sake of comparison the results obtained in the present study were compared with our previous results of the study of similar suspensions with human mesenchymal stem cells.

Both types of suspensions (with and without chitosan) enhance the secretion of cytokines by a 24-h culture of HBMLs (predominantly T-lymphocytes) compared to a control without nanoparticles. At a dose of 2.3, the MTD of chitosan promotes the stimulating effect on cells; in the dose range of MNPs 10–1000 MTDs, chitosan “inhibits” cellular secretory activity compared to “pure” MNPs. Both suspensions did not cause cell death by necrosis, hence, the secretion of cytokines is due to the enhancement of the functional activity of HBMLs. Increased accumulation of MNPs with chitosan in the cell fraction at 100 MTDs for 24 h exposure, according to energy dispersive X-ray fluorescence analysis, may be due to fixation of chitosan on the outer membrane of HBMLs. Within the cells, the distribution pattern of MNPs is similar.

The discussed results can be used for an addressed design of cell delivery and removal systems incorporating multiple activities and functions because of cell capability to avoid phagocytosis by immune cells as opposed to individual MNPs in the blood streem. They are also promising for the field of magnetic biosensor development. The possibility to incorporate living cells anchored with MNPs manipulation step using external magnetic field is also promising. 

## Figures and Tables

**Table 1:** Molecular determinants of HBMLs after 24 h of in vitro culture, Me (Q1–Q3).

% CD45^+^ Cells, Bearing Membrane Markers, *n* = 4 *								
CD3	CD4	CD8	CD25	CD95	CD28	CD71	RA	RO
99 (98–99)	67 (65–68)	21 (19–23)	9 (9–10)	15 (14–17)	79 (78–79)	2.5 (2–4)	55 (54–55)	35 (34–36)

* Note: *n* is the number of blood samples tested.

**Table 2:** Percentage of viable HBMLs (according to ISO 10993-5 test with 0.4% trypan blue) after 24-h co-cultivation with iron oxide MNPs from FF1 or FF2, Me (Q1–Q3).

Dose of MNPs in MTD	Amount of Viable Cells (%), *n* = 4 *	
	For FF1	For FF2
0	93.5 (88.5–95.5)	
2.3	95 (94.5–96)	95 (93–96)
10	94.5 (93.5–96)	93.5 (89–95)
100	95 (95–96)	94 (92.5–96.5)
1000	94.5 (93–97.5)	93.5 (91.5–96)

* Note: *n* is the number of samples tested in each group. Maximum tolerated dose (MTD) was 0.3 mg/L iron ions in water solution.

**Table 3:** The concentration of cytokines in supernatants of HBMLs after 24 h of co-cultivation with nanoparticles of iron oxides at various doses, Me (Q1–Q3).

MTD	The Concentration of Cytokines, pg/mL, *n* = 3							
	IL-2	IL-4	IL-6	IL-8	IL-10	GM-CSF	IFNγ	TNFa
0	4.23 (3.87–4.51)	1.26 (0.73–1.98)	49.0 (43.5–54.3)	2345 (2189–2416)	6.27 (5.99–8.55)	10.61 (10.23–13.40)	66.2 (59.1–68.9)	48.3 (44.3–56.4)
2.3 FF1	**6.50** **(6.23–6.56)**	1.82 (1.70–1.86)	**56.3** **(55.7–57.0)**	**3560** **(3507–3651)**	**14.28** **(13.2–15.32)**	**17.83** **(16.22–18.21)**	**97.6** **(95.4–100.2)**	**70.0** **(69.3–70.2)**
FF2	9.20 * **(9.03–10.11)**	2.65 * **(2.57–2.76)**	58.0 * **(57.7–58.5)**	4598 * **(4570–4610)**	17.56 * **(16-09–18-21)**	25.6 * **(24.2–26.0)**	137.7 * **(135.2–138.2)**	165.2 * **(152.3–167.4)**
10 FF1	**10.04** **(9.8–11.2)**	**2.92** **(2.82–3.01)**	**60.6** **(60.1–61.0)**	**4494** **(4489–4500)**	**52.74** **(48.12–56.33)**	**26.88** **(25.3–26.9)**	**146** **(132.8–147.5)**	**216.4** **(210.2–219.0)**
FF2	6.19 * (6.09–7.11)	1.85 * (1.8–1.95)	55.6 * (55.0–56.1)	2905 * (2800–3100)	10.4 * (10.18–11.1)	25.9 * (25.53–26.33)	110.1 * (108.2–112.6)	229.3 * **(220.3–230.3)**
100 FF1	**12.8** **(12.08–13.01)**	**3.42** **(3.32–3.56)**	**63.1** **(62.5–63.4)**	**4778** **(4770–4790)**	**267.6** **(259.3–280.9)**	**30.12** **(29.11–31.24)**	**197.7** **(189.0–2017)**	**278.3** **(262.0–280.7)**
FF2	3.8 * (3.63–4.01)	1.35 * (1.29–1.4)	50.7 * (50.0–51.0)	2900 * (2895–3000)	5.96 * (4.91–6.11)	16.92 * (16.8–17.22)	79.5 * (78.1–81.1)	78.3 * (76.2–81.8)
1000 FF1	**14.23** **(13.12–14.98)**	**3.82** **(3.75–4.01)**	**62.6** **(62.1–63.0)**	**4998** **(4980–5000)**	**295.5** **(285.3–302.3)**	**31.76** **(30.12–32.12)**	**200.9** **(198.9–202.2)**	**318.2** **(315.2–323.4)**
FF2	10.11 * (9.4–11.01)	2.98 * (2.81–3.01)	60.6 * (60.0–60.9)	5280 * **(5257–5310)**	60.8 * (58.2–61.1)	39.79 * **(37.8–40.22)**	161.6 * (159.8–162.1)	239.2 * (232.3–246.5)

Note: *n* is the number of samples tested in each group. Almost all values at doses 2.3–1000 MTD (bold font) are above the corresponding control samples (0 MTD); (*)—values for FF2 suspension are higher or lower than corresponding value for FF1 suspension without chitosan; *p* < 0.05 according to the Mann-Whitney test.

**Table 4:** The distribution of MNPs in the HBMLs cellular sediment and intercellular fluid (supernatants) according to energy dispersive X-ray fluorescence analysis, Me (Q1–Q3).

MTDs (ppm)	Cellular Sediments, *n* = 2–3		Supernatants, *n* = 3–4	
	For FF1	For FF2	For FF1	For FF2
0 (control)	0.6 (0.4–1.9); *n* = 3		1.45 (1.15–1.75); *n* = 4	
2.3 (0.69)	0.6–1.3	0.6–1.36	1.8 (1.2–2.6)	1.0 (0.15–1.2)
10 (3.0)	1.3	2.4–32	2.2 (1.2–3.0)	1.0 (0.9–5.3)
100 (30.0)	15.5–114.9	200–431	17.65 (15.95–19.10)	92.0 (45.7–97.0)
1000 (300.0)	32.8–38.8	307–408	43.25 (35.45–51.35)	5.7 (4.55–13.9)

Note: *n* is the number of samples tested in each group.
